# Immunohistochemical Study on the Expression of G-CSF, G-CSFR, VEGF, VEGFR-1, Foxp3 in First Trimester Trophoblast of Recurrent Pregnancy Loss in Pregnancies Treated with G-CSF and Controls

**DOI:** 10.3390/ijms21010285

**Published:** 2019-12-31

**Authors:** Fabio Scarpellini, Francesca Gioia Klinger, Gabriele Rossi, Marco Sbracia

**Affiliations:** 1 Center Endocrinology Reproductive Medicine (CERM), Viae Liegi 28, 00198 Rome, Italy; ananchekaityche@hotmail.com; 2 Histology and Embryology Section, Department of Biomedicine and Prevention, University of Rome, 00133 Rome, Italy; klinger@uniroma2.it (F.G.K.); gabrielereds@tiscali.it (G.R.)

**Keywords:** G-CSF, G-CSFR, VEGF, VEGFR-1 Foxp3, Treg, recurrent pregnancy loss

## Abstract

Background: Recurrent Pregnancy Loss (RPL) is a syndrome recognizing several causes, and in some cases the treatment with Granulocyte Colony Stimulating Factor (G-CSF) may be successful, especially when karyotype of the previous miscarriage showed no embryo chromosomal abnormalities. In order to evaluate the effects of G-CSF treatment on the decidual and trophoblast expression of G-CSF and its receptor, VEGF and its receptor and Foxp3, specific marker of putative Tregs we conducted an immunohistochemical study. Methods: This study was conducted on three groups of patients for a total of 38 women: in 8 cases decidual and trophoblast tissue were obtained from 8 women with unexplained RPL treated with G-CSF that miscarried despite treatment; in 15 cases the tissue were obtained from 15 women with unexplained RPL no treated; 15 cases of women who underwent voluntary pregnancy termination were used as controls. Tissue collected from these patients were used for immunohistochemistry studies testing the expression of G-CSF, G-CSFR, VEGF, VEGFR-1 and Foxp3. Results: G-CSF treatment increased the concentration of cells expressing Foxp3, specific marker for Tregs, in the decidua, whereas in no treated RPL a reduction of these cells was found when compared to controls. Furthermore, G-CSF treatment increased the expression of G-CSF and VEGF in the trophoblast. Conclusions: Our study showed that G-CSF treatment increased the number of decidual Treg cells in RPL patients as well as the expression of G-CSF and VEGF in villus trophoblast. These finding may explain the effectiveness of this treatment in RPL, probably regulating the maternal immune response through Tregs recruitment in the decidua, as well as stimulating trophoblast growth.

## 1. Introduction

Recurrent Pregnancy Loss (RPL) is currently described as the loss of two or more pregnancies prior to the 24th week of gestation [1]. It has been estimated that its frequency is 1 every 300 pregnant women [2]. The recognized causes for RPL are generally recognized parental chromosomal defects, infections, endocrinologic pathologies, uterine defects, antiphospholipid antibody, other autoimmune diseases, as well as embryo chromosomal abnormalities. However, more than 40% of RPL cases remain unexplained [3]. We have showed that in some of these patients the infusion of Granulocyte Colony Stimulating Factor (G-CSF) may be used successfully as a treatment especially in case the karyotyping of previous miscarriage showed no embryo chromosomal abnormalities [4].

G-CSF is a 177 aminoacid polypeptide with a molecular weight of about 25 Kd stimulating principally polymorphonuclear granulocytes [5] with a high affinity receptor known as G-CSFR or c-fms of 183 aminoacids with a molecular weight of approximately 14 Kd, present on the target cell surface [6]. In early papers, the expression of G-CSF was found on trophoblast cells and in decidual cells of several mammals including human placenta, as well as its receptor that was localized on the trophoblast cell surface [6,7,8,9,10,11,12,13]. The expression of G-CSF has been reported either only in the syncytio or in the cytotrophoblast, depending on the authors [8,9,12], as well as its for receptor, some authors reported its expression in the decidua and in the trophoblast in different pattern [8,9,12]. An anti-abortive role was also demonstrated for G-CSF in the animal model, and its absence was indirectly involved in early abortions [13,14,15]. It has also been shown in several in vitro studies and in animal models, that G-CSF has a positive effect on trophoblast growth and placenta metabolism [16,17].

Vascular endothelial growth factor (VEGF) is a glycoprotein with angiogenic properties [18,19] such as induction of vascular permeability [18,20,21], stimulation of endothelial cell division and migration [21,22] and in-vivo angiogenesis [18]. VEGF has three known receptors, but during the pregnancy, the VEGFR-1 or the fms-like tyrosine kinase (flt) is mainly expressed in the decidua and trophoblast cells during first trimester. VEGF was detected in cytotrophoblast during the first trimester, as well as its receptor, and within the decidua in first trimester pregnancy VEGF was found in the epithelial cells as well as VEGFR-1 [23,24,25]. Furthermore, it has been reported that in missed abortion of RPL women showed a reduced expression of VEGF and VEGFR-1 in trophoblast [26,27].

Foxp3-Treg cells are a subset of suppressive CD4 T helper cells essential for immune tolerance in humans and mice [28]. Several authors showed that in pregnancy there is a growth of peripheral blood Treg cell in both humans [29,30] and mice [31]. In these studies Treg cells showed to peak in the second trimester with a subsequent decrease through the delivery. Furthermore, it has been reported by several authors that in RPL women there is a reduction of Treg cells in the decidua and in the blood, suggesting a pivotal role for these immune cells in the pathogenesis of RPL [32,33,34].

In this study we evaluated the effects of G-CSF treatment on the maternal fetal interface using immunohistochemistry to assess the expression of G-CSF and its receptor, the VEGF and its receptor VEGFR-1, and Foxp3 in the trophoblast and decidua of first trimester miscarriages of RPL women treated with G-CSF that miscarried again despite the treatment, in no treated RPL and in normal first trimester pregnancies.

## 2. Results

### 2.1. Foxp3 Findings

**Figure 1:**
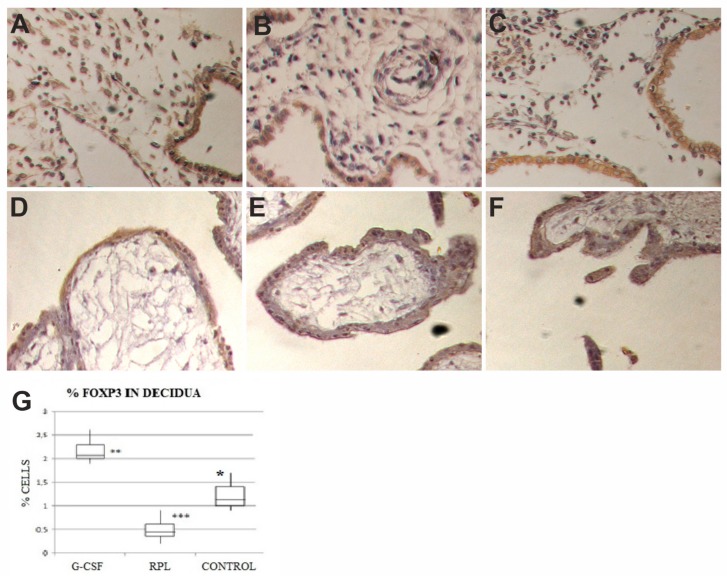
In decidua of Granulocyte Colony Stimulating Factor (G-CSF) treated samples (**A**), in the stroma there was a remarkable increase in the number of Foxp3 cells with respect to other two groups, epithelial cells showed a moderate staining (brown color) (400×). In decidua of no treated Recurrent Pregnancy Loss (RPL) samples (**B**), in the stroma there was a strong reduction in the number of Foxp3 cells, epithelial cells showed a moderate staining (brown color) (400×). In decidua of control samples (**C**), in the stroma there was a number of cells positive to Foxp3 cells, higher than in no-treated RPL and lower than in G-CSF treated samples, epithelial cells showed a moderate staining (brown color) (400×). The graph (**G**) showed percent of immunohistochemical positive cells in the stroma to Foxp3 (charts display median and quartiles with whiskers showing the range): There was a statistically significant difference between G-CSF vs. RPL (* *p* < 0.001), G-CSF vs. Control (** *p* < 0.01) and RPL vs. Control (*** *p* < 0.01). Foxp3 expression in decidua and trophoblast of first trimester pregnancy. In trophoblast of GCS-F treated group (**D**), no treated RPL (**E**) and Control (**F**) there was no staining at all for Foxp3 (400×).

In all three series of samples the syncytiotrophoblast and the cytotrophoblast did not show any specific staining for Foxp3, as well as villi stroma (Figure 1).

A weak staining for Foxp3 was found in the epithelial cells of the decidua of normal first trimester pregnancy (Figure 1), as well as in the epithelial cells of decidua in abortive pregnancies in women with RPL (Figure 1), as well as in the samples obtained from women treated with G-CSF (Figure 1), with a similar HSCORE values. In the stroma specific staining for Foxp3 was found in a relative small number of cells, putative Treg cells. Their number was lower in the samples obtained from RPL (0.4 ± 0.2), whereas was significantly higher in the samples obtained from RPL women treated with G-CSF (2.1 ± 0.6) with respect to controls (1.1 ± 0.3) (Figure 1B). These differences were statistically significant (*p* < 0.0001), (Figure 1)

No staining was found when primary antibody was incubated with a 10-fold molar excess of the antigen used for immunization. No differences were found among samples showing chromosomal abnormalities and samples with normal karyotype in both groups of G-CSF treated and no treated RPL, where the intensity of staining was consistent in all samples.

### 2.2. G-CSF and G-CSFR Findings

**Figure 2:**
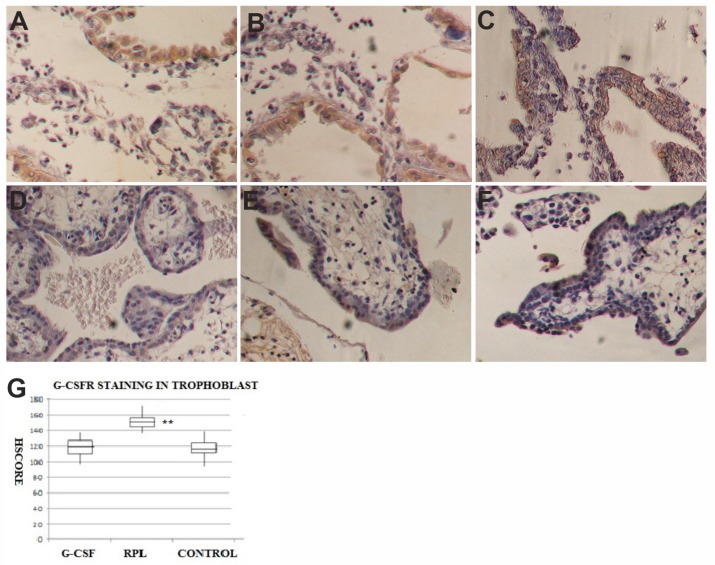
G-CSF expression in decidua and trophoblast of first trimester pregnancy. In decidua of G-CSF treated group (**A**), in the no treated RPL group (**B**) and Control pregnancies (**C**) there was the same staining levels in the epithelial cells but no in the stroma for G-CSF (brown color) (400×). In trophoblast of G-CSF treated samples (**D**), the syncytiotrophoblast was positive to G-CSF (brown color) (400×). In trophoblast of RPL no-treated samples (**E**), the syncytiotrophoblast was weakly positive to G-CSF (400×). In trophoblast of Control samples (**F**), the syncytiotrophoblast was positive to G-CSF (brown color) similar to G-CSF treated samples (400×). The graph (**G**) showed immunohistochemical staining semi quantitative HSCORE for G-CSF (Charts display median and quartiles with whiskers showing the range): There was statistically significant differences between G-CSF vs. RPL (* *p* < 0.001) and RPL vs. Control (** *p* < 0.001).

G-CSF was expressed in the epithelial cells of the decidua of normal first trimester pregnancy (Figure 2), as well as in the epithelial cells of decidua of abortive pregnancies in women with RPL, as well as in the samples obtained by women treated with G-CSF, with a similar intensity in HSCORE values. The stromal cells of the decidua showed no staining for G-CSF in all three series of samples.

The syncytiotrophoblast of the villi of normal first trimester pregnancies showed a strong staining (153 ± 44) for G-CSF (Figure 2), whereas in no treated RPL samples, a relevant reduction of staining for G-CSF (101 ± 36) (Figure 2) compared to the group of controls was found. In the samples obtained by women treated with G-CSF, the specific staining was similar to normal pregnancies (149 ± 46) (Figure 2). A statistical significant difference in HSCORE values (*p* < 0.001) was found among no treated RPL samples and the other two groups. In the cytotrophoblast G-CSF was expressed with a weaker intensity than in the syncytiototrophoblast in the three series of specimens with similar HSCORE values (Figure 2). The stroma of the villi in the three groups was negative for G-CSF.

**Figure 3:**
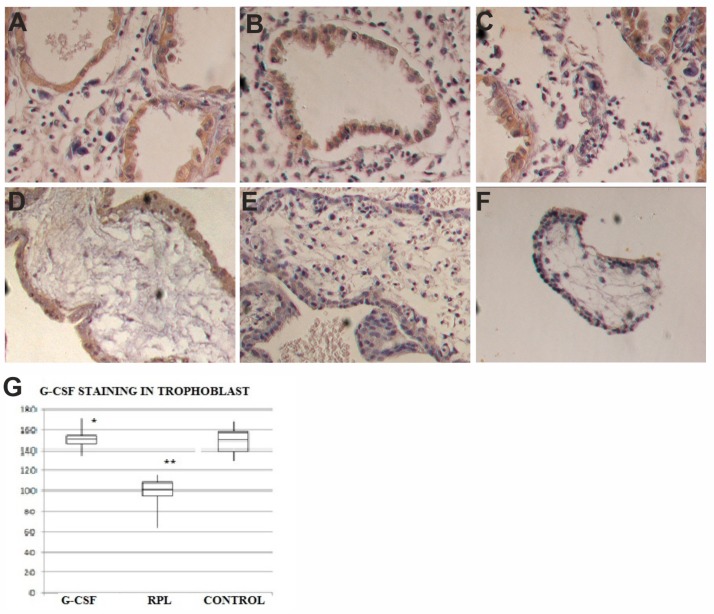
G-CSFR expression in decidua and trophoblast of first trimester pregnancy. In decidua of G-CSF treated group (**A**), in the no treated RPL group (**B**) and Control pregnancies (**C**) there was the same staining levels in the epithelial cells but no in the stroma for G-CSFR (brown color) (400×). In trophoblast of G-CSF treated samples (**D**), the syncytiotrophoblast was moderately positive to G-CSFR (brown colour) (400×). In trophoblast of RPL no-treated samples (**E**), the syncytiotrophoblast was strongly positive to G-CSFR, more than in other two groups (400×). In trophoblast of Control samples (**F**), the syncytiotrophoblast was moderately positive to G-CSFR (brown color) similar to G-CSF treated samples (400×). The graph (**G**) showed immunohistochemical staining semi quantitative HSCORE for G-CSFR (charts display median and quartiles with whiskers showing the range): There was a statistically significant differences between G-CSF vs. RPL (* *p* < 0.001) and RPL vs. Control (** *p* < 0.001).

The cytotrophoblast of villi of normal first trimester pregnancies was positive for G-CSFR (116 ± 39) (Figure 3), as well as in the specimens obtained by women treated with G-CSF (120 ± 41) (Figure 3). At the opposite, in the same cells of the villi in the RPL specimens there was a statistical significant increase in the specific staining for G-CSFR (150 + 38) (Figure 3) as shown in Figure 2 for the HSCORE (*p* < 0.001). In the syncytiotrophoblast, G-CSFR was expressed with weaker intensity than in the cytotrophoblast in all series of specimens with similar HSCORE values. The stroma of the villi in all three groups was negative for G-CSFR. No staining was observed when primary antibodies for G-CSF and G-CSFR were incubated with a 10-fold molar excess of the antigens used for immunization. No differences were found among samples showing chromosomal abnormalities and samples with normal karyotype in both groups of G-CSF treated and no treated RPL, where the intensity of staining was consistent in all samples.

### 2.3. VEGF and VEGF-R1 Findings

**Figure 4:**
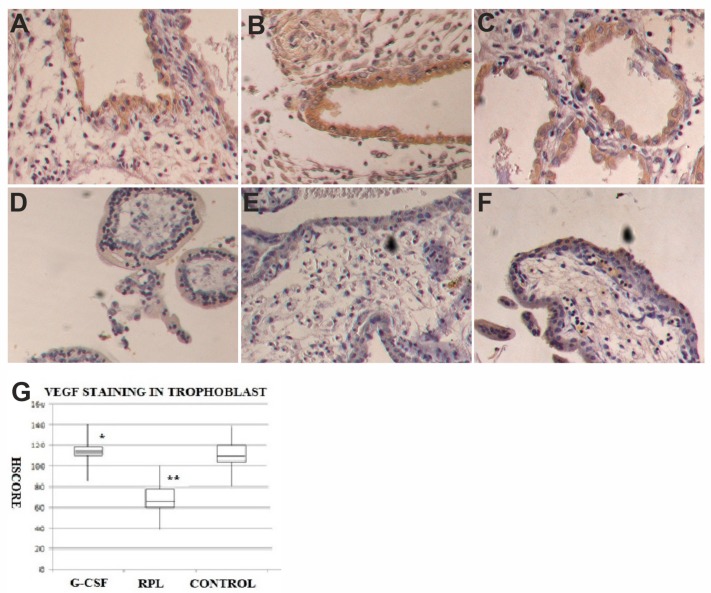
VEGF expression in decidua and trophoblast of first trimester pregnancy. In decidua of G-CSF treated group (**A**), in the no treated RPL group (**B**) and Control pregnancies (**C**) there was the same staining levels in the epithelial cells but no in the stroma for VEGF (brown color) (400×). In trophoblast of G-CSF treated samples (**D**), the syncytiotrophoblast was strongly positive to VEGF (brown color) (400×). In trophoblast of RPL no treated samples (**E**), the syncytiotrophoblast was weakly positive to VEGF (400×). In trophoblast of Control samples (**F**), the syncytiotrophoblast was strongly positive to VEGF (brown color) similar to G-CSF treated samples (400×). The graph (**G**) showed immunohistochemical staining semi quantitative HSCORE for VEGF (Charts display median and quartiles with whiskers showing the range): There was a statistically significant differences between G-CSF vs. RPL (* *p* < 0.001) and RPL vs. Control (** *p* < 0.001).

VEGF was expressed in the epithelial cells of the decidua of normal first trimester pregnancy (Figure 4A), as well as in the epithelial cells of decidua of abortive pregnancies in women with no treated RPL, as well as in the samples obtained by women treated with G-CSF, with a similar intensity in HSCORE values. The stromal cells of the decidua showed staining for VEGF in the endothelial cells of all three series of samples.

The syncytiotrophoblast of the villi of normal first trimester pregnancies showed positive staining for VEGF (108 + 32) (Figure 4), whereas in the same cells of no treated RPL specimens there was a relevant reduction of staining for VEGF (65 ± 21) (Figure 4). In the samples obtained by women treated with G-CSF, the specific staining for VEGF was similar in normal pregnancies (114 ± 36) (Figure 4). A statistical significant difference in HSCORE values (*p* < 0.001) was found among no treated RPL women samples and the other two groups (Figure 4). In the cytotrophoblast VEGF was expressed with a weaker intensity than in the syncytiototrophoblast in the three series of specimens with similar HSCORE values. The stroma of the villi showed a staining for VEGF in the vascular endothelial cells of all three groups of samples.

**Figure 5:**
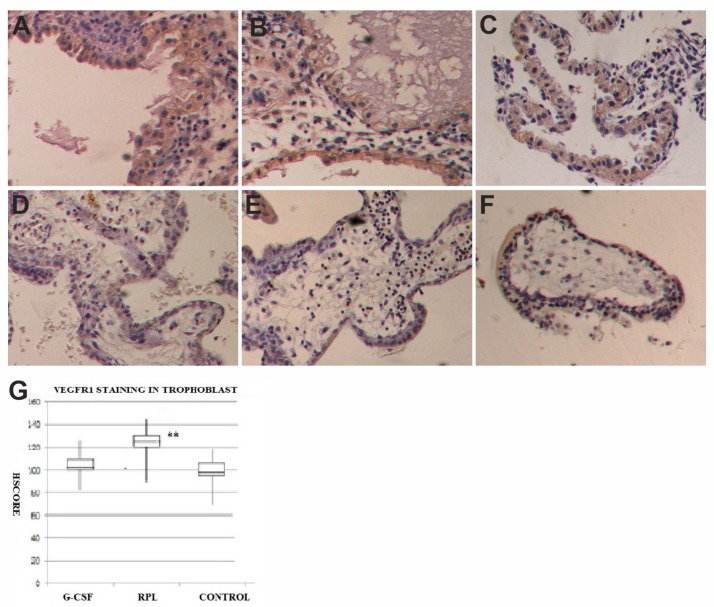
VEGFR-1 expression in decidua and trophoblast of first trimester pregnancy. In decidua of G-CSF treated group (**A**), in the no treated RPL group (**B**) and Control pregnancies (**C**) there was the same staining levels in the epithelial cells but no in the stroma for VEGFR-1 (brown color) (400×). In trophoblast of G-CSF treated samples (**D**), the syncytiotrophoblast was moderately positive to VEGFR-1 (brown color) (400×). In trophoblast of RPL no-treated samples (**E**), the syncytiotrophoblast was strongly positive to VEGFR-1, more than in other two groups (400×). In trophoblast of Control samples (**F**), the syncytiotrophoblast was moderately positive to VEGFR-1 (brown color) similar to G-CSF treated samples (400×). The graph (**G**) showed immunohistochemical staining semi quantitative HSCORE for VEGFR-1 (Charts display median and quartiles with whiskers showing the range): There was statistically significant differences between G-CSF vs. RPL (* *p* < 0.001) and RPL vs. Control (** *p* < 0.001).

The VEGFR-1 was expressed in the epithelial cells of decidua of normal first trimester pregnancies (Figure 5) as well as in the epithelial cells of decidua in abortive pregnancies in women with no treated RPL and in the samples obtained by women treated with G-CSF, with a similar intensity for the HSCORE. The stromal cells of decidua were mostly negative for VEGFR-1 in all three series of samples except that for vascular endothelial cells.

The cytotrophoblast of villi of normal first trimester pregnancies was positive for VEGFR-1 (99 ± 30) (Figure 5), as well as in the specimens obtained by women treated with G-CSF (104 ± 33) (Figure 5). At the opposite in the same cells of no treated RPL abortive specimens there was a statistical significant increase in the specific staining for VEGFR-1 (134 ± 37) (Figure 5B) for the HSCORE (*p* < 0.001). In the syncytiotrophoblast VEGFR-1 was expressed with weaker intensity than in the cytotrophoblast in all series of specimens with similar HSCORE values. The stroma of the villi in all groups was negative for VEGFR-1. No staining was observed when primary antibody was blocked by incubating the anti VEGF and anti VGFR-1 with a 10-fold molar excess of the antigens used in the immunization. No differences were found among samples showing chromosomal abnormalities and samples with normal karyotype in both groups of G-CSF treated and no treated RPL, where the intensity of staining was consistent in all samples.

Furthermore, when a linear regression analysis was performed among the levels of G-CSF staining and the number of Foxp3 positive cells in the stroma of decidua, a statistical significant positive correlation was found (r = 0.89 “*p* < 0.001”). Similarly a statistical significant positive correlation was found (r = 0.87 “*p* < 0.001”), when a regression analysis was conducted among levels of G-CSF staining and the values of staining for VEGF in the trophoblast, as well as when the regression analysis was conducted between G-CSFR staining and VEGFR-1 staining values (r = 0.81; “*p* < 0.001”).

## 3. Discussion

We previously reported the successful use of G-CSF in the treatment of unexplained RPL [4]. In this immunohistochemical study the effects of G-CSF treatment were evaluated in first trimester decidua and trophoblast of miscarried pregnancies despite the treatment compared to normal pregnancies and no treated RPL miscarriages.

Our data showed that in the decidua of pregnancies treated with G-CSF there was a significant increase of Foxp3 positive stromal cells, putative Treg cells, when compared to the other two groups. It is well known that Treg cells play an essential role in reproduction, as demonstrated by evidence in human and in animal models. These cells seem involved in all process of reproduction, from the implantation to parturition, and several data showed their dysregulation may be involved in infertility, RPL, preeclampsia and preterm labor. In endometrium there is a recruitment of Treg cells starting in the proliferative phase of the cycle, with an estrogen dependent manner [35]. After increasing in early pregnancy, decidual Tregs remain elevated during mid gestation [29]. These cells are composed both of thymus derived Tregs, positive to the Helios antigen, an Ikaros transcription factor family member, and peripheral originated Tregs negative for the Helios antigen [36]. The peripheral generated Tregs play a fundamental role in maternal-fetal tolerance, since they seem determine the expansions of Treg population in early pregnancy decidua [37], whereas thymic Tregs may preferentially recruited in first trimester decidua [38,39]. However, other studies reported that Helios is a marker for activation, proliferation and immunosuppressive activity of Tregs instead than a marker of thymic origin [40]. Decidual Tregs secrete IL-10, and TGF-β and express CD25, CTLA4 and PD-L1, all markers of Treg suppression activity: they promote anti-inflammatory and tolerogenic phenotype in macrophages, dendritic cells and NK cells [41]. It has been reported that in preeclampsia Tregs are reduced in both decidua and maternal peripheral blood [42,43,44], as well as their suppression function is impaired with a concomitant rise of proinflammatory Th17 cells [30,45,46]. This seems due to a preferential reduction of peripheral originated Tregs, especially in early onset severe preeclampsia [44]. In primarily unexplained infertility, a reduced endometrial expression of FoxP3 has been reported [47], as well as successful IVF cycles seem associated with an increase of Tregs in the peripheral blood [48]. It is known that G-CSF can modulate immune response in inflammatory diseases, mobilize Treg cells from bone marrow and induce Tregs production [49,50]. All these data taken into account together may explain, in part, the effects of G-CSF treatment in RPL. The administration of G-CSF induces Treg cells development as well as their mobilization from bone marrow that move to decidua, with the increase of their number as we observed in RPL cases, where they were decreased when no treated with G-CSF.

RPL is a syndrome that may recognize several possible causes, in particular chromosomal abnormalities of embryos account for a large portion of miscarriages, also in unexplained RPL. Several authors claimed that in RPL there was a reduced number of Tregs in decidua [32,51,52,53], as well as in maternal blood [35,53,54]. Only two papers pointed out the role of chromosomal abnormalities, differentiating decidua from miscarriages with abnormal or normal embryo karyotype. Inada et al. in 2013 [55] described that the frequency of Tregs in decidua of miscarriages with normal embryo karyotype was significantly lower than in normal progressing pregnancies and in miscarriages with an abnormal karyotype embryos. Furthermore, Ki67 negative Treg population was smaller in normal chromosomic embryo miscarriages than normal pregnancies, even though Ki67 positive Treg population was similar in all three groups. In a subsequent paper of 2015 the same group showed that Helios positive Treg were decreased in decidua of miscarriages with normal chromosomic embryos with respect to normal progressing pregnancies and in the miscarriages with an abnormal karyotype [39]. Our data showed a strong increase of decidual positive FoxP3 cells in women treated with G-CSF, miscarrying again despite treatment, compared to no treated RPL cases and controls. Furthermore, in no treated RPL cases decidual positive FoxP3 cells were decreased also with respect to controls. Contrarily to such reported by Inada et al. in their two papers of 2013 and 2015 [39,55], we did not observe any differences between the miscarriages with normal or abnormal karyotype. This diversity in findings may due to the differences in techniques used as well as by the fact that these patients were treated with G-CSF, which seems increase the levels of these cells. Furthermore, we did not test our samples for Helios antigen, although the real role of this antigen in differentiating the Treg sub-populations remains to be clarified. These findings, whether confirmed, may represent an important new insight in the reproductive immunology, revealing a direct connection between G-CSF and the recruitment of Treg into the decidua or their local induction.

Recently, Nadkarni et al. [56] showed that neutrophils may activate Treg, inducing in naïve T cells of decidua the expression of Foxp3, and promoting the secretion of IL-10, IL-17 and VEGF in these cells. The neutrophil induced Tregs show proangiogenic activity on decidual tissue and trophoblast. G-CSF treatment in RPL women sensibly increases neutrophils blood levels (neutrophils count over than 15,000/mL), this is the normal and therapeutic response to G-CSF administration. The neutrophils so stimulated may further explain the Foxp3 cells increase observed in the decidua of women treated with G-CSF. Furthermore, we observed a concomitant increase of G-CSF and VEGF positivity in the villi trophoblast of the pregnancy treated with G-CSF, correlating with the levels of Foxp3 cells in decidua. These findings may be due other than to a direct G-CSF stimulation of trophoblast, also to the proangiogenic function of Treg induced through neutrophils activated by G-CSF treatment.

In our study on first trimester trophoblast villi, the G-CSF was found mainly in the syncytiotrophoblast and with less intensity in staining in the cytotrophoblast. Instead, the G-CSF receptor was expressed primarily in the cytotrophoblast of chorionic villi, and with lower intensity in the syncytiotrophoblast. In addition to this, G-CSF and its receptor were found in the epithelial cells of decidua, according to earlier data published by several authors [6,7,8,9,10,11,12]. Our findings showed that the syncytiotrophoblast of RPL no treated specimens had a statistically significant decrease in G-CSF expression with respect to the controls and G-CSF treated cases, whereas its receptor was over expressed in the cytotrophoblast of RPL with respect to the controls and G-CSF treated women. In a recent study, Rahamati et al. [57] showed that in secretory endometrium of implantation failure patients there was a decrease in G-CSFR mRNA expression with respect to recurrent miscarriage cases and controls, and when the endometrial biopsy were “in vitro” cultured with G-CSF the mRNA of G-CSFR increased. These data, obtained from no pregnant samples and after in vitro culture of the tissue, substantially confirm our findings since they showed the effects of G-CSF supplementation on endometrium. The authors did not report where was observed the increase of G-CSFR mRNA, in epithelial or stromal components, whereas our data were obtained in an “in vivo” study on samples obtained from pregnant women. Rahmati et al. [57] in their study observed that the expected role of G-CSF supplementation should be demonstrated on “in vivo” study, and in some way, our study may answer to the questions raised from the authors.

The G-CSF system seems to play a regulatory role in the trophoblast growth and development: an earlier study showed that trophoblast cells incubated with G-CSF showed a significant increase of cell proliferation [11]. Furthermore, the supplementation with G-CSF of trophoblast cell line culture promotes cell growth by MAP Kinase activation with a reduction of apoptosis in the trophoblast and increased cell survival [17].

Furthermore, to evaluate the possible effect of G-CSF treatment on the trophoblast development we tested the expression of VEGF and its receptor VEGFR-1. Even though we did not find relevant differences in the expression of these two antigens in the decidua of the three groups, at the opposite the trophoblast of RPL patients showed a significant reduction in the staining for VEGF, whereas controls and RPL treated with G-CSF showed a good VEGF specific staining, mainly observed in syncytiotrophoblast. At the same time, the VEGFR-1 specific staining resulted significantly increased the cytotrophoblast of no treated RPL specimens. In controls and G-CSF treated pregnancies, the same levels of VEGFR-1 specific staining were observed. It is well known that VEGFR-1 is the receptor mainly expressed in trophoblast throughout pregnancy, and in the past, several authors reported the possible involvement of VEGF system in miscarriage [26,27]. Our data in particular seem to agree with such reported by Vuorela et al. [26], showing a reduction of VEGF expression in trophoblast. Furthermore, a striking positive correlation was found between G-CSF and VEGF expression in the syncytiotrophoblast and between G-CSFR and VEGFR-1 in the cytotrophoblast. This observation is particularly interesting since no treated RPL miscarriages showed a concomitant reduction of G-CSF and VEGF expression with respect to control pregnancies; instead, in G-CSF treated miscarriages, we observed staining levels overlapping the levels of controls, suggesting that G-CSF treatment may recover the normal trophoblast growth and development, as well as a direct effect on VEGF expression. Instead G-CSFR and VEGFR-1 showed a concomitant increase in no treated RPL miscarriage trophoblast; these data suggested that the reduction of G-CSF expression may promote the over expression of its receptor in target cells. This effect seems to be reversed by G-CSF treatment, since we observed that G-CSFR and VEGFR-1 staining levels in samples obtained from G-CSF treated women were close to normal pregnancies. These data, taken in account together, suggest that G-CSF play a fundamental role during implantation and trophoblast invasion in the first trimester pregnancy. G-CSF promotes at fetal-maternal interface the increase of immune suppressor T cells, and at the same time with an autocrine/paracrine mechanism it stimulates trophoblast growth and development such as suggested by VEGF and VEGFR-1 system correlated to G-CSF activity.

A questionable point of our study is the use of only immunohistochemistry, without genes expression analysis using Real Time PCR Our study was an “in vivo” morphological analysis for the expression of several antigens evaluated in different tissues, decidua with stromal and epithelial component, and trophoblast, with syncytium, cyto and stroma, villi components. Gene expression analysis using mRNA extracted from these tissues is not suitable for the scopes of our study. The tissues collected after a dilatation and curettage procedure abortions are a bloody pulp constituted from blood, decidua and trophoblast villi, where only after fixation and inclusion is possible to recognize the different tissues. The extraction of mRNA from these samples cannot allow the differentiation of its origin, losing the information from which cells it originated and in which amount it is produced. The isolation of the different types of cells and their in “vitro” culture allow to define the real origin of the mRNA, but it is another story. In this study we want to evaluate the “in vivo” effects of G-CSF treatment in RPL pregnant women on decidua and trophoblast, and up to date the only technique available and reliable remains immunohistochemistry.

However, our findings should be taken with caution until they are confirmed from further study, since it may be a consequence of embryo demise and blood flow reduction in the trophoblast or its initial re-absorption.

Our study showed that G-CSF and its receptor may play a relevant role in the growth and development of the trophoblast during the first trimester of pregnancy and they may be involved in the mechanisms determining RPL. Furthermore, the use of G-CSF in the treatment of RPL seems play a role in regulating the maternal immune response at the embryo’s antigens promoting Treg recruitment, and at same time stimulating the trophoblast growth.

## 4. Materials and Methods

This immunohistochemical study was conducted on tissue specimens of first trimester pregnancies obtained after dilatation and curettage (D&C) from a total of 38 pregnant women. They were divided in three groups: eight women with RPL treated with G-CSF that miscarried again despite the treatment, 15 women with RPL that miscarried during the clinical screening and did not receive any treatment and 15 healthy women underwent voluntary pregnancy termination used as controls. All women with RPL history (G-CSF treated and without treatment) underwent at the same clinical screening to exclude known causes of RPL (the patients were tested for parental karyotype, uterine structure, endocrinological anomalies included diabetes, immunological tests including anticardiolipin antibodies and lupus anticoagulant, congenital thrombophilia, etc.). All patients had to be negative for all these tests for study inclusion. Furthermore, patients treated with G-CSF in their previous miscarriage had to show a normal embryo karyotype, mandatory condition to be treated with G-CSF. All the pregnancies included in this study were spontaneous pregnancies, since pregnancies obtained by ART were not included. The tissue obtained from D&C of all RPL patients miscarrying again were karyotyped by array-CGH to know the chromosomal status of embryos.

A total of 8 cases with unexplained RPL (age 33.1 ± 2.0) were treated with recombinant G-CSF with a daily subcutaneous administration of Filgastrim (Neupogenor, Dompe or Granulokine 30, Amgen, Italy), at a dosage of 1 mg (100,000 IU)/kg/day from the sixth day after ovulation until the occurrence of abortion [4]. These miscarriages showed an abnormal karyotype in six cases whereas two were normal.

The fifteen miscarriages of no treated women with unexplained RPL (age 31.6 + 2.3), showed in 8 cases an abnormal karyotype, whereas in 7 cases the karyotype was normal.

Controls tissue specimens were obtained by 15 terminated voluntarily pregnancies from the 8th through the 12th week of gestation with the presence of fetal heart beat. These women had no history of previous abortions and they were 30.8 + 2.2 years old. Demographic data of the patients and controls are reported in Table 1.

All surgical procedures were carried out in the Center of Endocrinology and Reproductive Medicine (CERM) Rome, Italy. The project had the approval of the Institutional Ethical Committee (CH-F2017, released 14-4-2017). All patients signed an informed written consent for the use of their tissue samples

Tissue samples were fixed in formalin at 4 °C overnight and subsequently paraffin embedded. Each sample of decidua and trophoblast were fixed without separation of embryonic and decidua tissues in order to have both trophoblastic villi and decidua on the same slide. Serial sections of the same samples 5 µm thick were used for immunohistochemistry. Commercially available monoclonal antibodies were used for the detection of G-CSF and G-CSFR (Santa Cruz Biotechnology, Dallas, TX, USA), VEGF and VGFR-1 (Santa Cruz Biotechnology), and Foxp3 (Santa Cruz Biotechnology).

Immunohistochemistry was performed on dewaxed and rehydrated sections, and endogenous peroxidase quenching was obtained with 0.3% hydrogen peroxide in methanol for 30 min incubation at room temperature. All slides were incubated for a non-immune block with normal horse serum for 30 min at room temperature before the incubation with the first antibody, carried out at room temperature for 1 h with a dilution of 1 to 100 for the all monoclonal mouse antibodies. Thereafter tissue sections were labeled with an avidin-biotin-peroxidase detection system ABC Vectastain (Vector Laboratories, Burlingthon, VT, USA). Each step was followed by three washing with phosphate buffer saline solution. Finally, 3,3’-diaminobenzidine was used as a chromogen. A light counterstaining was performed with Meyer’s hematoxylin with only 30 s bath in order to observe the eventual nuclear staining for the specific antigens. Controls were performed using the G-CSF antibody, G-CSFR antibody, VEGF antibody, VEGFR-1 antibody, Foxp3 antibody, preabsorbed overnight with a tenfold excess molar excess of the respective immunogen peptides purchased by the same Company (Santa Cruz Biotechnology, Dallas, TX, USA). Furthermore, slides were incubated with nonimmune rabbit IgG at equal concentration as further control.

For a semi-quantitative analysis of specific staining the HSCORE system was used according to the following equation: HSCORE = ∑Pi(i + 1), where i is the intensity of staining with a value of 1, 2, or 3 (weak, strong or very strong respectively) and Pi is the percentage of cells stained for each intensity, varying from 0% to 100%. Ten microscopic fields were counted by two of the authors independently for each slide (MS and FS). The intra-observer and inter-observer variation coefficient were 3.4% and 4.2% respectively. At least 3 slides of each sample were observed, for both antigens tested, under the microscope and each observer was blind to which sample it was (RPL or control slides). The slides, numbered progressively, were blindly analyzed by the two different observers and scored for HSCORE, and each value was recorded on the worksheet according to the corresponding patient for the given number. The HSCORE analysis was performed separately for the villous trophoblast, cytotrophoblast and syncytiotrophoblast, and the decidua, both component glandular and stromal cells (each observer performed 4 different HSCOREs for each slide). For Foxp3 quantification ten medium power fields were selected to count Foxp3 positive cells, the results was given in percent of total stromal decidua cells.

Statistical analysis. Differences between groups were determined using the Mann-Whitney U test. Significance was determined as *p* < 0.05. All statistical analyses were performed with SPSS 17.0.

## Figures and Tables

**Table 1:** Demographic data of three groups of patients in the study. (Data reported in mean ± SD).

	G-CSF	RPL	Controls
Number of patients	8	15	15
Age: when pregnancy started	33.1 ± 3.0	31.6 ± 2.3	30.8 ± 2.2
BMI: when pregnancy started	27.7 + 2.1	27.4 ± 1.9	27.8 ± 1.8
Smokers (more than 10 cigarette per day)	0	1	2
Number of previous abortions	5.6 + 0.7	5.5 ± 0.4	0
Gestational week of miscarriage (range)	7.9 ± 1.3 (5–9)	8.1 ± 1.2(5–9)	9.4 ± 1.1(7–10)
